# Maternal Coffee Consumption During Pregnancy and Attention-Deficit/Hyperactivity Disorder in Offspring: A Case–Control Study and Meta-Analysis

**DOI:** 10.3390/ijerph22121808

**Published:** 2025-11-30

**Authors:** Ahmed Arafa, Amira S. A. Said, Ehab Elkady, Tarig A. Y. Ali, Doaa Mahmoud Khalil

**Affiliations:** 1 Department of Preventive Cardiology, National Cerebral and Cardiovascular Center, Suita 564-8565, Japan; 2 Department of Public Health, Faculty of Medicine, Beni-Suef University, Beni-Suef 62521, Egypt; doaamahmoud@med.bsu.edu.eg; 3 Department of Clinical Pharmacy, College of Pharmacy, Al Ain University, Al Ain 64141, United Arab Emirates; amira.ahmed@aau.ac.ae; 4 Ada’a Health Center, Ministry of Health, Riyadh 11451, Saudi Arabia; eelkady@moh.gov.sa (E.E.); tabali@moh.gov.sa (T.A.Y.A.)

**Keywords:** ADHD, caffeine, coffee, neurodevelopment, pregnancy, lifestyle, prevention, risk factors

## Abstract

Background: Attention-deficit/hyperactivity disorder (ADHD) is a common neurodevelopmental disorder. Maternal diet can influence fetal neurodevelopment, and coffee is widely consumed during pregnancy and may have adverse effects on fetal development. This study aimed to investigate the association between maternal coffee consumption during pregnancy and ADHD risk in offspring. Methods: First, we conducted a case–control study in Egypt, enrolling 176 mothers of children with ADHD and 504 mothers of typically developing children. ADHD was diagnosed according to the Diagnostic and Statistical Manual of Mental Disorders, Fifth Edition (DSM-5). Multivariable logistic regression was used to estimate odds ratios (ORs) and 95% confidence intervals (CIs) for ADHD associated with frequent maternal coffee consumption during pregnancy. Then, we combined the results of this case–control study with those from prior studies in a meta-analysis. Between-study heterogeneity was assessed using the *I*^2^ statistic, and publication bias was evaluated by Egger’s regression test. Results: In the case–control study, frequent maternal coffee consumption during pregnancy was associated with a higher risk of ADHD in offspring (OR = 1.85; 95% CI: 1.17, 2.92). This association persisted after additional adjustments for antenatal, natal, and neonatal factors (OR = 1.82; 95% CI: 1.07, 3.09). Consistently, the meta-analysis showed a higher risk of ADHD associated with maternal coffee consumption during pregnancy (n = 7, OR = 1.33; 95% CI: 1.13, 1.57), with no between-study heterogeneity (*I*^2^ = 8.89%, *p* = 0.36) or publication bias (z = 0.10, *p* = 0.92). Conclusions: Both our case–control study and meta-analysis suggest that higher maternal coffee consumption during pregnancy may increase the risk of ADHD in children. Still, prospective cohort studies with objective caffeine biomarkers are needed to clarify causality and determine safe exposure levels.

## 1. Introduction

Attention-deficit/hyperactivity disorder (ADHD) is one of the most prevalent neurodevelopmental disorders in childhood, affecting about 8% of children globally [1,2]. ADHD is characterized by developmentally inappropriate levels of inattention, hyperactivity, and impulsiveness, which often lead to poorer school performance, social difficulties, and long-term functional impairment [2,3]. Beyond its individual impact, ADHD contributes to 0.8% of all disability-adjusted life years attributable to mental disorders globally, imposing substantial societal costs through educational support needs, productivity losses, and increased healthcare spending [4,5].

The etiology of ADHD is multifactorial, involving a complex interaction of genetic, environmental, and neurobiological factors [6]. Maternal exposures during critical periods of fetal brain development may trigger epigenetic modifications that increase the risk of ADHD in offspring [7,8]. Among these exposures, maternal nutritional behaviors during pregnancy have been associated with both fetal neurodevelopment and ADHD pathogenesis [9,10].

Caffeine, the most widely consumed psychoactive substance globally, has drawn particular attention in this context. During pregnancy, caffeine is rapidly absorbed, crosses the placenta, and is metabolized more slowly due to reduced cytochrome P450 activity. Because the fetus lacks sufficient enzymes to metabolize caffeine, accumulation may occur, potentially disrupting neuronal proliferation, synaptogenesis, and sleep-wake regulation [11]. Accumulating evidence indicates that maternal caffeine consumption during pregnancy is associated with an increased risk of adverse pregnancy and fetal outcomes, including miscarriage, stillbirth, low birth weight and/or small for gestational age (SGA), and childhood obesity and acute leukemia [10,11,12]. Therefore, the American College of Obstetricians and Gynecologists advises that pregnant women limit their caffeine intake to <200 mg/day (about 2 cups of coffee) to prevent adverse pregnancy outcomes [13]. Nevertheless, caffeine consumption among pregnant women remains common and frequently exceeds these limits. For example, data from the Finnish Kuopio Birth Cohort showed that 31.2% of women consumed >200 mg/day of caffeine during the first trimester, rising to 38.2% during the third trimester [14]. In the French EDEN Mother–Child Cohort, 12% of pregnant women consumed ≥200 mg/day of caffeine [15]. The Japan Environment and Children’s Study reported that 16.5% of pregnant women consumed >300 mg/day of caffeine [16].

Clinical studies have demonstrated that excessive prenatal caffeine exposure alters fetal brain morphology and behavioral development. The Adolescent Brain and Cognitive Development study showed that children, aged 9–10 years, who were exposed to caffeine in utero had lower MRI fractional anisotropy values, an indicator of white matter integrity, in the left inferior fronto-occipital fasciculus and left corticospinal tract compared to those with minimal exposure. They also exhibited higher scores on measures of psychopathology, including inattention, hyperactivity, and daydreaming, indicating a greater risk of neurodevelopmental problems [17]. Nishihara et al. found that maternal caffeine intake exceeding 300 mg/day during pregnancy, compared to <100 mg/day, was associated with 1.11-fold higher odds of gross motor developmental delay in children at 12 months [16]. Galéra et al. reported that children of mothers consuming ≥200 mg/day of caffeine had a higher likelihood of borderline or lower IQ at 5.5 years compared with those whose mothers consumed < 100mg/day [15].

However, epidemiological evidence on the association between maternal coffee or caffeine consumption during pregnancy and the risk of ADHD in offspring is inconsistent [18,19,20,21,22,23,24,25,26]. In a Korean case–control study involving parents of 2673 children, including those with full-syndrome and subthreshold ADHD, no significant association between maternal caffeine consumption and ADHD was found, with odds ratios (ORs) of 1.28 (95% confidence intervals (CIs): 0.81, 2.02) for full-syndrome ADHD and 1.04 (0.73, 1.49) for subthreshold ADHD [20]. A cohort study from the Netherlands that assessed child behavior using the Strengths and Difficulties Questionnaire also reported no significant association between maternal caffeine consumption and ADHD symptoms, with ORs ranging between 0.87 and 1.08 across increasing exposure categories [18]. Similarly, a Brazilian birth cohort with 11 years of follow-up found no association between maternal caffeine consumption and ADHD, with ORs (95% CIs) of 1.12 (0.68, 1.84) for moderate (100–299 mg/day) and 0.90 (0.51, 1.59) for high (≥300 mg/day) caffeine consumption [21]. Findings from the Norwegian Mother, Father and Child Cohort (MoBa) study indicated no significant association between caffeine consumption up to 300 mg/day and ADHD-related outcomes, with ORs ranging from 0.87 to 1.13 across exposure levels [24]. Using data from the same study, a Mendelian randomization analysis observed a positive association between maternal coffee consumption during pregnancy and offspring inattention/hyperactive-impulsive behavior; however, this association became null after further adjustments [25]. Polygenic risk score (PRS) analyses, involving data from the UK Avon Longitudinal Study of Parents and Children, the Dutch Generation R study, and the Norwegian MoBa study, showed no association between coffee consumption during pregnancy and maternal-reported ADHD symptoms [26]. In contrast, results from the Danish National Birth Cohort showed that consuming ≥ 8 cups/day of coffee during the first trimester was significantly associated with a 47% higher risk of behavioral disorders consistent with ADHD [22]. Another large Danish cohort indicated a dose-dependent but statistically non-significant increase in ADHD risk with higher maternal coffee consumption, with relative risks (RRs) ranging from 0.9 (95% CI: 0.5, 1.6) for 1–3 cups/day to 2.3 (95% CI: 0.9, 5.9) for ≥10 cups/day [19]. Collectively, the epidemiological evidence remains inconclusive, likely reflecting methodological differences in exposure measurement, outcome assessment, confounder adjustment, and cultural patterns of coffee consumption.

Importantly, most available evidence originates from Western countries, leaving significant geographical gaps in regions such as North Africa and the Middle East, where dietary patterns and beverage preferences may differ [27]. In these regions, caffeine consumption is culturally common. In Egypt, for example, caffeinated beverages are heavily consumed, with a mean caffeine intake of 255 mg/day (2.5 cups of coffee), and over one-third of the population exceeds the safe limit of 3 mg/kg body weight/day [28], set by the European Food Safety Authority [29]. Despite this, few studies have assessed caffeine consumption patterns among pregnant women in Egypt. A cross-sectional study from Port Said in northern Egypt reported that 28.4% of women consumed coffee during pregnancy [30], while another study from Minia in southern Egypt found that 95% of pregnant women regularly consumed coffee or tea [31]. Neither study assessed the proportion of women who exceeded the recommended safe caffeine level during pregnancy.

No studies to date have examined the association between maternal coffee consumption and ADHD risk in Egypt or the Middle East. Furthermore, no prior meta-analysis has synthesized the global evidence on this association. We hypothesized that frequent maternal coffee consumption during pregnancy would be associated with an increased risk of ADHD in offspring. Therefore, we conducted a case–control study, using data from Egypt, to examine the association between maternal coffee consumption during pregnancy and ADHD risk in offspring. Then, we integrated the findings of the Egyptian study with existing global evidence through a systematic review and meta-analysis to obtain a more generalizable conclusion.

## 2. Materials and Methods

### 2.1. The Case–Control Study

#### 2.1.1. Study Population

A total of 176 mothers of children diagnosed with ADHD were recruited from the outpatient clinics of Beni-Suef University Hospital between November 2019 and January 2021. This hospital is a major teaching institution serving as the primary referral center for residents of Beni-Suef Governorate in southern Egypt, providing specialized psychiatric, speech, phoniatric, and dietetic services for children with neurodevelopmental disorders. Eligible cases were mothers of children aged 4–13 years who had been clinically diagnosed with ADHD. The mean age ± standard deviation (SD) of children with ADHD was 9.2 ± 3.4 years. For the control group, invitation letters were distributed in March 2020 to mothers of children attending a public primary school with a kindergarten located near Beni-Suef University Hospital. Mothers who agreed to participate received a self-administered questionnaire. Eligible controls were mothers of neurotypical children aged 4–13 years with no history of ADHD or other psychiatric or neurodevelopmental disorders (e.g., autism spectrum disorder, intellectual disability, or epilepsy). To minimize potential recall bias, control participants were instructed to provide information regarding their youngest child. Mothers were excluded if they were unable to recall pregnancy-related information. Eventually, the control group consisted of 504 mothers of neurotypical children. The mean age ± SD of children in the control group was 6.5 ± 1.9 years. The same control group was used in a previous study [32].

#### 2.1.2. Ascertainment of Outcome, Exposure, and Covariates

*Outcome:* Based on the criteria outlined in the Diagnostic and Statistical Manual of Mental Disorders, Fifth Edition (DSM-5) [33], ADHD was diagnosed when a persistent pattern of inattention and/or hyperactivity-impulsivity interfered with functioning or development. This required the presence of at least six symptoms from either the inattention or hyperactivity-impulsivity domains, persisting for at least six months to a degree inconsistent with the individual’s developmental level, causing a direct negative impact on social, academic, or occupational activities. ADHD diagnoses were either made directly by psychiatrists at Beni-Suef University Hospital or verified by them for children referred from other facilities.

*Exposure:* Maternal coffee consumption during pregnancy was assessed using a self-administered questionnaire. Participants were asked to indicate how often they consumed coffee during pregnancy, with response options of “rarely or never,” “once/day,” and “>once/day.” Because only a few participants reported drinking coffee > once/day, the latter two categories were combined into a single group labeled “frequent consumption,” whereas “rarely or never” was classified as “infrequent consumption.” Information on the type of coffee (e.g., caffeinated, decaffeinated, or instant) was not collected.

*Covariates:* Information on potential confounding variables, including maternal, antenatal, natal, and neonatal characteristics, was obtained using the same self-administered questionnaire. Maternal personal factors included residence (urban or rural), employment status (working or not), educational level (none/elementary or higher), and consanguineous marriage. Antenatal and natal variables comprised maternal age at pregnancy (<35 or ≥35 years), exposure to passive smoking, gestational diabetes, gestational hypertension, threatened abortion, and mode of delivery (vaginal or cesarean section). Neonatal characteristics included the child’s sex, birth weight (low or normal), presence of respiratory distress, and admission to the neonatal intensive care unit (NICU). The questionnaire was developed by the investigators based on previous epidemiological studies on maternal and perinatal risk factors for neurodevelopmental disorders [18,19,20,21,22,23,24]. Its content validity was reviewed by two specialists in public health and psychiatry to ensure clarity and relevance. Since the questionnaire items assessed factual variables (e.g., coffee consumption, personal information, and pregnancy complications), rather than psychometric constructs, internal consistency reliability was not evaluated.

#### 2.1.3. Statistical Analysis

The chi-squared test was used to compare maternal and neonatal characteristics between cases and controls. Subsequently, logistic regression analysis was conducted to estimate ORs and 95% CIs for the association between frequent maternal coffee consumption during pregnancy and ADHD in offspring. Logistic regression was chosen because the outcome variable (ADHD diagnosis) was binary, and this method enables adjustment for multiple potential confounders. The associations were presented sequentially: Model I adjusted for maternal personal factors, Model II additionally adjusted for antenatal and natal factors, and Model III further adjusted for neonatal factors. We performed statistical analysis using the Statistical Package for Social Science (SPSS) (IBM SPSS Statistics for Windows, Version 22.0, IBM Corporation, Armonk, NY, USA).

### 2.2. The Meta-Analysis

#### 2.2.1. Registration

This meta-analysis was conducted per the guidelines of the Preferred Reporting Items for Systematic Reviews and Meta-Analyses (PRISMA) [34]. The study protocol was prospectively registered with the International Prospective Register of Systematic Reviews (PROSPERO; ID: CRD420251126325).

#### 2.2.2. Eligibility Criteria

Studies were considered eligible for inclusion if they satisfied the following criteria: ADHD in offspring was the outcome; maternal caffeine/coffee consumption during pregnancy was the exposure; and the study reported risk estimates (e.g., ORs or RRs) or incidence/prevalence data of ADHD across categories of caffeine or coffee consumption. We excluded duplicates, case reports, animal studies, and reviews.

#### 2.2.3. Search Strategy

We conducted a systematic literature search in the Medline (PubMed), Web of Science, and Scopus databases to identify relevant studies published before 1 August 2025, without restrictions on publication year. In addition, the reference lists of the included articles and relevant reviews were manually screened to identify additional eligible studies. Two authors independently conducted the literature search, study selection, and data extraction using predefined keywords and eligibility criteria (Table A1). Titles and abstracts were first screened for relevance, followed by a full-text review of potentially eligible studies. For each included study, data were extracted on the first author’s name, year of publication, study location, sample size, study design, categories of maternal caffeine/coffee consumption, methods of ADHD assessment, covariates included in the adjusted models, and corresponding risk estimates with 95% CIs. Any discrepancies between the two authors were resolved through discussion. The extracted information was further cross-checked against the original articles before analysis.

#### 2.2.4. Quality Assessment

The methodological quality of the included studies was evaluated using a modified form of the Newcastle–Ottawa Scale (NOS) [35]. The criteria assessed included the definition and representativeness of cases and controls, comparability between study groups, the method used to ascertain maternal coffee intake during pregnancy, uniformity of data collection procedures across groups, and participant response rates.

#### 2.2.5. Statistical Analysis

A random-effects model was employed to estimate the pooled ORs and 95% CIs of ADHD in offspring, comparing the highest and lowest categories of maternal coffee consumption during pregnancy [36]. Heterogeneity across studies was assessed using τ^2^ (total heterogeneity), *I*^2^ (proportion of total variability due to heterogeneity), and H^2^ (ratio of total variability to sampling variability) statistics [37]. Publication bias was assessed using Egger’s regression test and visual inspection of funnel plots [38]. Subgroup analyses were conducted based on study design (cohort vs. case–control), population (Western vs. non-Western), and ADHD ascertainment method (DSM vs. other diagnostic criteria). To evaluate whether associations differed significantly across subgroups, we compared pooled ORs using a z-test for heterogeneity. Sensitivity analyses were conducted by excluding studies one by one and combining the remaining studies in separate meta-analyses. All statistical analyses were conducted using the R 4.5.1 statistical software package (Metafor: Meta-Analysis Package for R) [39].

## 3. Results

### 3.1. The Case–Control Study

Compared with controls, women with ADHD children showed higher proportions of urban residence (64.2% vs. 35.5%, *p* < 0.001), consanguineous marriage (38.1% vs. 15.7%, *p* < 0.001), and passive smoking during pregnancy (60.8% vs. 25.8%, *p* < 0.001). Gestational diabetes (8.0% vs. 1.8%, *p* < 0.001), gestational hypertension (5.7% vs. 1.4%, *p* = 0.004), and threatened abortion (16.5% vs. 3.6%, *p* < 0.001) were also more common among women of children with ADHD. Male sex predominated in cases (76.1% vs. 44.0%, *p* < 0.001), along with higher rates of respiratory distress (11.9% vs. 7.1%, *p* = 0.038) and admission to NICU (35.2% vs. 13.7%, *p* < 0.001) (Table 1).

In the model adjusted for maternal personal factors, frequent maternal coffee consumption during pregnancy was associated with a higher risk of ADHD in offspring (OR = 1.85; 95% CI: 1.17, 2.92). This association remained robust after further adjustment for antenatal, natal, and neonatal factors (OR = 1.82; 95% CI: 1.07, 3.09) (Table 2).

### 3.2. The Meta-Analysis

**Figure 1:**
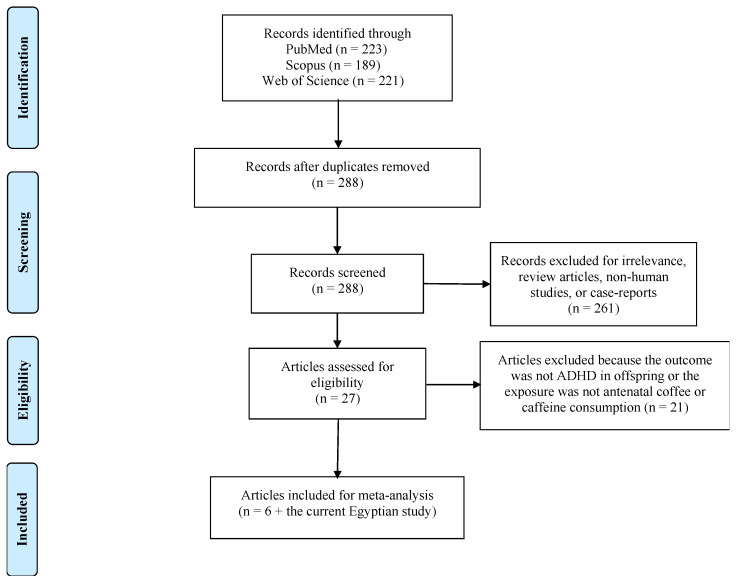
PRISMA flowchart of the studies included in the meta-analysis.

A total of 7 studies (6 previously published studies in addition to the current case–control study), including 98,295 mothers, were included in the meta-analysis (Figure 1).

Of these, 5 employed a cohort design: Linnet et al. [19], Loomans et al. [18], Del-Ponte et al. [21], Hvolgaard Mikkelsen et al. [22], and Berglundh et al. [24], while Kim et al. [15] and Arafa et al. used a case–control design. The studies were conducted in Korea, Denmark, the Netherlands, Brazil, Norway, and Egypt. ADHD diagnosis was based on DSM criteria in 4 studies, and most adjusted for multiple confounders, including child sex, maternal lifestyle behaviors and medical conditions, and sociodemographic characteristics (Table 3). According to the modified NOS, all included studies were rated as being of moderate to high quality (Table 4).

**Figure 2:**
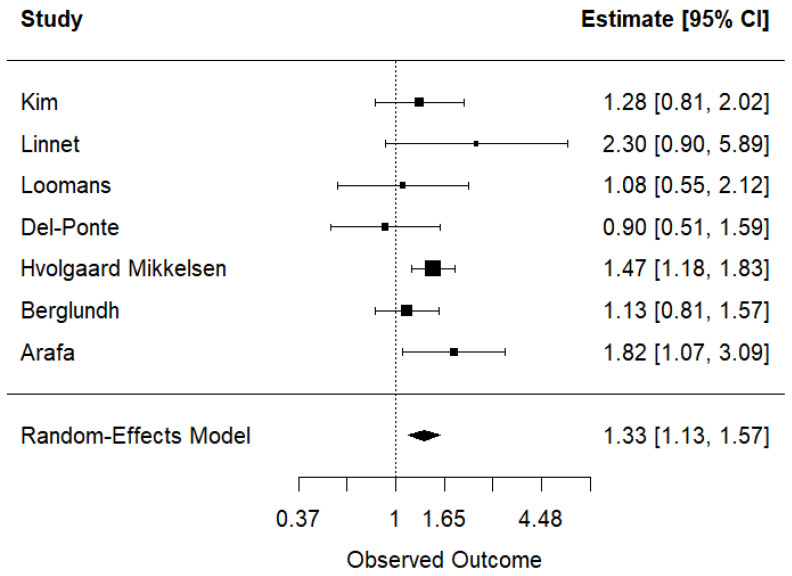
Meta-analysis of the association between maternal coffee consumption during pregnancy and attention-deficit/hyperactivity disorder in offspring [18,19,20,21,22,24].

The relative contribution of each study to the pooled meta-analysis was as follows: Kim et al. (11.9%) [20], Linnet et al. (3.0%) [19], Loomans et al. (5.7%) [18], Del-Ponte et al. (7.9%) [21], Hvolgaard Mikkelsen et al. (40.8%) [22], Berglundh et al. (21.6%) [24], and Arafa et al. (9.1%). Only Hvolgaard Mikkelsen et al. [22] and Arafa et al. reported statistically significant positive associations between maternal coffee consumption during pregnancy and ADHD in offspring, whereas the remaining studies showed non-significant associations. Overall, the meta-analysis showed a significant association between maternal coffee consumption during pregnancy and ADHD risk in offspring (OR = 1.33; 95% CI: 1.13, 1.57), with no evidence of heterogeneity (τ^2^ = 0.01, *I*^2^ = 8.89%, H^2^ = 1.10; *p* = 0.36) (Figure 2).

**Figure 3:**
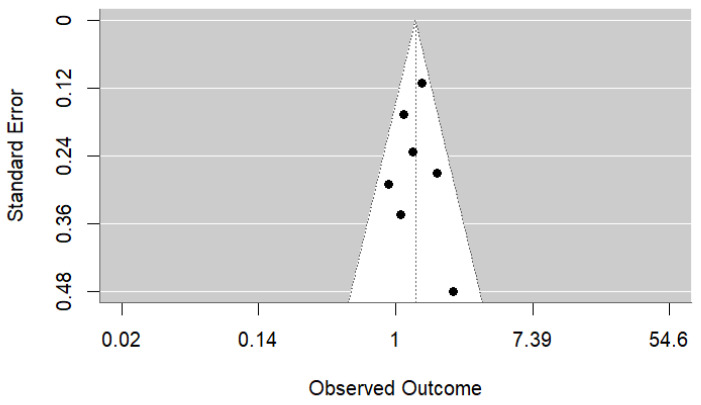
Funnel plot of the studies investigating the association between maternal coffee consumption during pregnancy and attention-deficit/hyperactivity disorder in offspring.

No publication bias was detected (Figure 3), and Egger’s regression test for funnel plot asymmetry was non-significant (z = 0.10, *p* = 0.92).

When stratified by study design, the association remained statistically significant for both cohort studies (OR = 1.28; 95% CI: 1.03, 1.58; *I*^2^ = 23.18%) and case–control studies (OR = 1.49; 95% CI: 1.05, 2.01; *I*^2^ = 0.00%). The associations did not significantly differ by region (ORs = 1.35 in Western populations vs. 1.29 in non-Western populations) or by assessment methods (ORs = 1.39 in DSM-diagnosed ADHD vs. 1.33 with other diagnoses) (Table 5). The z-tests for heterogeneity were > 0.40 in all subgroup comparisons, indicating no statistically significant differences between subgroups.

Sequentially removing each study and re-running the meta-analysis did not materially alter the overall association. The pooled ORs (95% CIs) ranged from 1.25 (1.01, 1.55) after excluding Hvolgaard Mikkelsen et al. [22] to 1.40 (1.17, 1.67) after excluding Berglundh et al. [24]. Similarly, between-study heterogeneity remained largely unchanged (Table A2).

## 4. Discussion

### 4.1. The Case–Control Study

This study demonstrated a positive association between higher maternal coffee consumption during pregnancy and the risk of ADHD in offspring. The association remained significant after adjusting for several maternal and neonatal factors, suggesting an independent relationship.

Of note, we detected a higher risk of ADHD associated with consuming coffee ≥one cup/day (almost ≥100 mg/day of caffeine), suggesting that maternal coffee consumption during pregnancy may be harmful even at levels below the current recommended limit. Alike, previous studies reported adverse fetal outcomes when maternal caffeine intake was <200 mg/day. For instance, the Norwegian MoBa study showed that caffeine consumption within safe limits was associated with an increased risk of SGA births [40]. Alike, in the Finnish Kuopio Birth Cohort, the women with moderate caffeine consumption (51–200 mg/day) during the first trimester were at risk of having a SGA newborn [14]. A prospective cohort study, using data from the US Kaiser Permanente Medical Care Program, showed a dose-response relationship between caffeine intake, including levels < 200 mg/day, and fetal growth restriction [41]. In the Japan Environment and Children’s Study, moderate caffeine consumption (125.5–205.5 mg/day) was associated with SGA and preterm birth in the second trimester [42]. Therefore, maternal coffee consumption during pregnancy should be approached with caution, as potential complications may occur at levels below current guidelines.

This study had many strengths. It investigated an understudied population with unique cultural factors and dietary habits, drew on a large sample, applied standardized ADHD diagnostic criteria, and adjusted the results for multiple potential confounding factors. Nonetheless, several limitations warrant consideration. First, coffee consumption was assessed retrospectively through self-report, which might have introduced recall bias and precluded establishing causal relationships. Second, the study population may have been skewed toward more severe cases of ADHD, as cases were recruited from mothers of children who visited clinics for ADHD symptoms. Third, due to the limited availability of cases and the relative feasibility of recruiting controls, we adopted an approximately 1:3 case-to-control ratio to improve statistical power. Fourth, we did not collect information on mothers’ habitual coffee consumption before pregnancy or during lactation. We also did not collect information about the family history of neurodevelopmental disorders. Fifth, we did not collect data on coffee consumption by trimester. Hvolgaard Mikkelsen et al. [22] reported that the impact of maternal coffee intake on ADHD risk was greater when consumed during the first trimester compared to the third trimester. Sixth, merging the maternal coffee consumption categories of “once/day” and “>once/day” into a single group due to the limited number of respondents in the latter category prevented us from assessing a dose–response association. Seventh, data on antenatal and natal factors included in the regression models were collected through a self-administered questionnaire rather than hospital records; therefore, recall and misclassification biases are possible. Eighth, the absence of ADHD among the control group children was not clinically confirmed. Since ADHD is often underdiagnosed [43], some controls may have had undetected symptoms, potentially leading to misclassification bias. Ninth, we did not assess other sources of caffeine intake or quantify the total amount of caffeine consumed.

### 4.2. The Meta-Analysis

This meta-analysis is, to our knowledge, the first to quantitatively synthesize evidence on the association between maternal coffee consumption during pregnancy and ADHD risk. Consistent with the case–control study, higher levels of caffeine intake during pregnancy were associated with an elevated risk of ADHD in offspring. Most included studies were of moderate to high quality, adjusted for multiple confounders, and used standardized criteria for ADHD diagnosis. The analysis showed low heterogeneity, consistent findings across subgroups, and no evidence of publication bias. Moreover, the results remained stable across all sensitivity analyses, including leave-one-out analyses, indicating that no single study disproportionately influenced the overall effect.

Previous meta-analyses have consistently reported adverse fetal outcomes associated with maternal caffeine consumption during pregnancy. A meta-analysis of 22 studies found that caffeine consumption was linked to a 28% higher risk of SGA births [44]. Another meta-analysis of seven studies reported a 70% increase in the risk of low birth weight [45]. Additionally, a meta-analysis of 14 studies showed that caffeine consumption was associated with an elevated risk of pregnancy loss, with risk increases of 40% for high consumption (350–699 mg/day) and 72% for very high consumption (≥700 mg/day) [46]. In a meta-analysis of seven studies, consuming coffee during pregnancy was associated with a 39% rise in the risk of preeclampsia, a major risk factor for fetal complications [47]. Taken together with our findings, these meta-analyses highlight the potential vulnerability of fetal growth and development to maternal caffeine exposure and reinforce the importance of monitoring caffeine consumption during pregnancy to minimize the risk of adverse fetal outcomes.

Noteworthy, our meta-analysis had several limitations. First, the limited number of studies included in this meta-analysis reduced the statistical power of subgroup and sensitivity analyses. Second, the geographical coverage of the studies was limited, which may reduce the generalizability of the findings to other populations with different lifestyles or dietary habits. Third, there were differences in how maternal coffee consumption during pregnancy was measured, including recall periods, serving size definitions, and whether caffeine content was assessed, which may have caused exposure misclassification. Fourth, ADHD diagnosis methods varied between studies, ranging from parental reports to clinical assessments, which may have contributed to outcome variation. Fifth, although most studies adjusted their results for major confounders, residual confounding from unmeasured factors such as maternal diet cannot be ruled out.

### 4.3. Clinical and Public Health Implications

From a clinical perspective, this study highlights the importance of incorporating caffeine and coffee consumption screening into routine antenatal assessments. Early identification of high-risk consumption patterns would allow healthcare providers to deliver personalized counseling and reduce potential neurodevelopmental risks to the fetus. From a public health standpoint, it underscores the need to revisit the guidelines on caffeine intake during pregnancy. Policymakers could also consider enforcing clearer labeling of caffeine content in beverages to promote informed decision-making among expectant mothers.

### 4.4. Future Research

Future investigations should use large, prospective cohort designs with repeated measures of caffeine and coffee intake throughout the pregnancy trimesters. Incorporating biochemical markers of caffeine exposure, such as serum or urinary caffeine and paraxanthine concentrations, would reduce recall bias. Further research should also explore dose–response associations and examine potential threshold effects to identify safe levels of caffeine intake for pregnant women. Cross-cultural comparative studies would also help determine whether cultural differences in beverage type and preparation method influence fetal outcomes. From a mechanistic perspective, neuroimaging follow-up studies could provide insight into how maternal caffeine exposure affects brain development, attention regulation, and cognitive functioning.

## 5. Conclusions

This study indicated that maternal coffee consumption during pregnancy was associated with an increased risk of ADHD in offspring, independent of major maternal and neonatal factors. From a clinical perspective, these findings highlight the need to assess caffeine intake routinely during antenatal care and provide counseling to reduce excessive consumption. From a public health perspective, implementing clear labeling of caffeine content on packaged drinks, integrating nutrition education into antenatal programs, and developing national dietary guidelines for pregnancy would collectively support informed decision-making. Furthermore, large prospective cohort studies are warranted to confirm these associations, explore dose–response relationships, and elucidate biological pathways.

## Figures and Tables

**Table 1:** Characteristics of mothers of children with attention-deficit/hyperactivity disorder and their controls (the case–control study).

Characteristics		Cases n = 176	Controls n = 504	*p*-Value
Maternal personal factors				
Residence, %	Rural	35.8	64.5	<0.001
	Urban	64.2	35.5	
Job, %	Housewife	68.8	69.8	0.428
	Working	31.3	30.2	
Education, %	Basic	54.0	48.8	0.137
	Higher	46.0	51.2	
Married to a relative, %	No	61.9	84.3	<0.001
	Yes	38.1	15.7	
Maternal antenatal and natal factors				
Pregnancy age, %	<35 years	86.9	89.3	0.236
	≥35 years	13.1	10.7	
Exposure to passive smoking, %	No	39.2	74.2	<0.001
	Yes	60.8	25.8	
Gestational diabetes, %	No	92.0	98.2	<0.001
	Yes	8.0	1.8	
Gestational hypertension, %	No	94.3	98.6	0.004
	Yes	5.7	1.4	
Threatened abortion, %	No	83.5	96.4	<0.001
	Yes	16.5	3.6	
Delivery method, %	Vaginal	22.2	26.8	0.133
	C–section	77.8	73.2	
Neonatal factors				
Sex, %	Female, %	23.9	56.0	<0.001
	Male, %	76.1	44.0	
Low-birth weight, %	No	75.6	80.4	0.109
	Yes	24.4	19.6	
Respiratory distress, %	No	88.1	92.9	0.038
	Yes	11.9	7.1	
Admission to the neonatal intensive care unit, %	No	64.8	86.3	<0.001
	Yes	35.2	13.7	

**Table 2:** The association between maternal coffee consumption during pregnancy and attention-deficit/hyperactivity disorder in offspring (the case–control study).

	Maternal Coffee Consumption During Pregnancy	
	Infrequent	Frequent
Cases, %	71.6	28.4
Controls, %	84.3	15.7
Model I, OR (95% CI)	1 (Reference)	1.85 (1.17, 2.92)
Model II, OR (95% CI)	1 (Reference)	1.90 (1.15, 3.12)
Model III, OR (95% CI)	1 (Reference)	1.82 (1.07, 3.09)

Model I: Adjusted for maternal personal factors; Model II: Model I + antenatal and natal factors; and Model III: Model II + neonatal factors.

**Table 3:** Summary of the studies included in the meta-analysis.

Study ID	Study Design	Population	ADHD Assessment	Coffee/Caffeine Categories and Results	Covariates
Kim et al. (2009) [20] Korea	Case–control	Parents of 123 children with full syndrome ADHD, 231 with subthreshold ADHD, and 2319 without ADHD	DSM-IV	Caffeine (yes or no) ORs (95% CIs) = 1.28 (0.81, 2.02) for full syndrome ADHD and 1.04 (0.73, 1.49) for subthreshold ADHD	Age, child sex, and socioeconomic status
Linnet et al. (2009) [19] Denmark	Cohort	24,068 women from the Aarhus Birth Cohort at 16 gestational weeks and their children were followed for 12 years	DSM-IV	Coffee (0, 1–3, 4–9, or ≥10 cups/day) RRs (95% CIs) = 0.9 (0.5, 1.6), 1.3 (0.7, 2.3), and 2.3 (0.9, 5.9), respectively	Smoking, alcohol, maternal age, cohabitant status, child sex, education, employment, and parental and siblings’ psychiatric status
Loomans et al. (2012) [18] Netherlands	Cohort	3439 women and their children at the age of 5	Strengths and Difficulties Questionnaire (SDQ)	Caffeine (0–85, 86–255, 256–425, or >425 mg/day) ORs (95% CIs) = 0.94 (0.68, 1.31), 0.87 (0.57, 1.33), and 1.08 (0.55, 2.12), respectively	Age, ethnicity, education, family size, cohabitant status, alcohol, smoking, anxiety, child sex, childbirth weight, and gestational age
Del-Ponte et al. (2016) [21] Brazil	Cohort	3485 newborns followed for 11 years	DSM-IV	Caffeine (<100, 100–299, or ≥300 mg/day) ORs (95% CIs) = 1.12 (0.68, 1.84) and 0.90 (0.51, 1.59), respectively	Maternal mood symptoms during pregnancy, paternal education, National Economic Index, and maternal conjugal situation
Hvolgaard Mikkelsen et al. (2017) [22] Denmark	Cohort	46,531 women from the Danish National Birth Cohort at 15 gestational weeks and their children were followed for 11 years	Strengths and Difficulties Questionnaire (SDQ)	Coffee (0, 0.5–3, 4–7, or ≥8 cups/day) RRs (95% CIs) = 0.97 (0.88, 1.08), 1.09 (0.93, 1.27), and 1.47 (1.18, 1.83), respectively	Child sex, birth year, maternal age, parity, socioeconomic status, maternal body mass index, and tea drinking
Berglundh et al. (2021) [24] Norway	Cohort	15,819 full-term pregnancies from the Norwegian Mother, Father and Child Cohort Study and their children were followed for 8 years	Screen for child anxiety-related disorders (SCARED)	Caffeine (0–22, 23–56, 57–200, 201–300, or >300 mg/day) ORs (95% CIs) = 0.97 (0.81, 1.15), 0.87 (0.74, 1.03), 1.05 (0.81, 1.36), and 1.13 (0.81, 1.57), respectively	Maternal age, alcohol, smoking, marital status, child sex, maternal education, household income, dietary fiber, total energy intake, nausea, and maternal mental health
Arafa et al. (2025) Egypt (this study)	Case–control	176 mothers of children diagnosed with ADHD at one university hospital and 504 control mothers	DSM-5	Coffee (>once/day, once/day, or never/rarely) OR (95% CI) = 1.82 (1.07, 3.09) for ≥once/day versus never/rarely	Residence, employment, education, consanguinity, gestational age, passive smoking, diabetes, hypertension, threatened abortion, delivery method, child sex, and neonatal complications (low birth weight, respiratory distress, and admission to NICU)

**Table 4:** Quality assessment of the studies included in the meta-analysis using the Newcastle–Ottawa Scale.

**Items**	**Linnet** **et al. [19]**	**Loomans** **et al. [18]**	**Del-Ponte** **et al. [21]**	**Hvolgaard Mikkelsen** **et al. [22]**	**Berglundh** **et al. [24]**
*Cohort studies*					
Representativeness of the exposed group	*	*	*	*	*
Method used to determine exposure	*	*	*	*	*
Selection process for the unexposed group	*	*	*	*	*
The outcome was absent at the baseline	*	*	*	*	*
Comparability	*	*	*	*	*
Method used to assess outcomes	*	*	*	*	*
Follow-up long enough for outcomes to occur	*	*	*	*	*
Completeness and adequacy of follow-up	*	--	*	*	--
Overall (total number of asterisks *)	8	7	8	8	7
**Items**	**Kim et al. [20]**	**Arafa et al.**			
*Case–control studies*					
Definition and clarity of case identification	*	*			
Representativeness of the selected cases	*	*			
Method of control selection	*	*			
Definition of the control group	*	*			
Comparability	*	*			
Assessment of exposure	--	*			
Consistency of exposure assessment between cases and controls	*	*			
Evaluation of nonresponse rate	--	--			
Overall (total number of asterisks *)	6	7			

**Table 5:** Meta-analysis of the association between maternal coffee consumption during pregnancy and attention-deficit/hyperactivity disorder in offspring stratified by study design, population, and assessment method.

Factors		Number of Studies	OR (95% CI)	*I* ^2^
Study design	Cohort	5	1.28 (1.03, 1.58)	23.18%
	Case–control	2	1.49 (1.05, 2.10)	0.00%
Population	Western	4	1.35 (1.11, 1.64)	10.68%
	Others	3	1.29 (0.89, 1.88)	36.67%
Assessment	DSM	4	1.39 (0.97, 1.97)	32.61%
	Others	3	1.33 (1.10, 1.60)	5.39%

## Data Availability

Data can be made available from the corresponding author upon reasonable request after seeking the approval of the Research Ethics Committee of Beni-Suef University.

## Abbreviations

The following abbreviations are used in this manuscript:

ADHD	Attention-deficit/hyperactivity disorder
OR	Odds ratio
CI	Confidence interval
DSM	Diagnostic and Statistical Manual of Mental Disorders
MoBa	Mother, Father, and Child Cohort Study
PROSPERO	International Prospective Register of Systematic Reviews
NICU	Neonatal intensive care unit
NOS	Newcastle-Ottawa Scale
PRISMA	Preferred Reporting Items for Systematic Reviews and Meta-Analyses
RR	Relative risk
SD	Standard deviation
SGA	Small for gestational age

## Appendix A

**Table A1:** Search Strategy.

**Search** (Coffee OR Caffeine OR Beverages) AND (ADHD) (“coffee”[Supplementary Concept] OR “coffee”[All Fields] OR “coffee”[MeSH Terms] OR “coffee s”[All Fields] OR “coffees”[All Fields] OR (“caffein”[All Fields] OR “caffeinated”[All Fields] OR “caffeine”[Supplementary Concept] OR “caffeine”[All Fields] OR “caffeine”[MeSH Terms] OR “caffeine s”[All Fields] OR “caffeines”[All Fields] OR “caffeinism”[All Fields]) OR (“pregnancy”[MeSH Terms] OR “pregnancy”[All Fields] OR “pregnancies”[All Fields] OR “pregnancy s”[All Fields])) AND (“attention deficit disorder with hyperactivity”[MeSH Terms] OR (“attention”[All Fields] AND “deficit”[All Fields] AND “disorder”[All Fields] AND “hyperactivity”[All Fields]) OR “attention deficit disorder with hyperactivity”[All Fields] OR “adhd”[All Fields])
**Translations** Coffee: “coffee”[Supplementary Concept] OR “coffee”[All Fields] OR “coffee”[MeSH Terms] OR “coffee’s”[All Fields] OR “coffees”[All Fields] Caffeine: “caffein”[All Fields] OR “caffeinated”[All Fields] OR “caffeine”[Supplementary Concept] OR “caffeine”[All Fields] OR “caffeine”[MeSH Terms] OR “caffeine’s”[All Fields] OR “caffeines”[All Fields] OR “caffeinism”[All Fields] pregnancy: “pregnancy”[MeSH Terms] OR “pregnancy”[All Fields] OR “pregnancies”[All Fields] OR “pregnancy’s”[All Fields] ADHD: “attention deficit disorder with hyperactivity”[MeSH Terms] OR (“attention”[All Fields] AND “deficit”[All Fields] AND “disorder”[All Fields] AND “hyperactivity”[All Fields]) OR “attention deficit disorder with hyperactivity”[All Fields] OR “adhd”[All Fields]

**Table A2:** Sensitivity analyses.

Removed Study	OR (95% CI)	*I* ^2^
Kim et al. [20]	1.33 (1.09, 1.63)	23.52%
Linnet et al. [19]	1.32 (1.12, 1.55)	5.60%
Loomans et al. [18]	1.35 (1.12, 1.62)	18.87%
Del-Ponte et al. [21]	1.38 (1.19, 1.62)	0.00%
Hvolgaard Mikkelsen et al. [22]	1.25 (1.01, 1.55)	7.04%
Berglundh et al. [24]	1.40 (1.17, 1.67)	4.50%
Arafa et al. (this study)	1.30 (1.11, 1.53)	4.16%
